# Polymorphisms in B Cell Co-Stimulatory Genes Are Associated with IgG Antibody Responses against Blood–Stage Proteins of *Plasmodium vivax*

**DOI:** 10.1371/journal.pone.0149581

**Published:** 2016-02-22

**Authors:** Gustavo C. Cassiano, Adriana A. C. Furini, Marcela P. Capobianco, Luciane M. Storti-Melo, Maristela G. Cunha, Flora S. Kano, Luzia H. Carvalho, Irene S. Soares, Sidney E. Santos, Marinete M. Póvoa, Ricardo L. D. Machado

**Affiliations:** 1 Department of Biology, São Paulo State University (Universidade Estadual Paulista - UNESP), São José do Rio Preto, state of São Paulo (SP), Brazil; 2 Department of Dermatologic, Infectious, and Parasitic Diseases, College of Medicine of São José do Rio Preto, São José do Rio Preto, SP, Brazil; 3 Department of Biology, Federal University of Sergipe, Aracaju, Sergipe, Brazil; 4 Laboratório of Microbiology and Immunology, Institute of Biological Sciences, Federal University of Pará (Universidade Federal do Pará - UFPA), Belém, state of Pará (PA), Brazil; 5 Laboratory of Malaria, René Rachou Research Center, Oswaldo Cruz Foundation, Belo Horizonte, state of Minas Gerais (MG), Brazil; 6 Department of Clinical and Toxicological Analyses, Faculty of Pharmaceutical Sciences, University of São Paulo (Universidade de São Paulo - USP), São Paulo, SP, Brazil; 7 Laboratory of Human and Medical Genetics, Federal University of Pará, Belém, PA, Brazil; 8 Laboratory of Basic Research in Malaria, Section of Parasitology, Evandro Chagas Institute, Belém, PA, Brazil; Agency for Science, Technology and Research - Singapore Immunology Network, SINGAPORE

## Abstract

The development of an effective immune response can help decrease mortality from malaria and its clinical symptoms. However, this mechanism is complex and has significant inter-individual variation, most likely owing to the genetic contribution of the human host. Therefore, this study aimed to investigate the influence of polymorphisms in genes involved in the costimulation of B-lymphocytes in the naturally acquired humoral immune response against proteins of the asexual stage of *Plasmodium vivax*. A total of 319 individuals living in an area of malaria transmission in the Brazilian Amazon were genotyped for four SNPs in the genes *CD40*, *CD40L*, *BLYS* and *CD86*. In addition, IgG antibodies against *P*. *vivax* apical membrane antigen 1 (PvAMA–1), Duffy binding protein (PvDBP) and merozoite surface protein 1 (PvMSP–1_19_) were detected by ELISA. The SNP *BLYS* –871C>T was associated with the frequency of IgG responders to PvAMA–1 and PvMSP–1_19_. The SNP *CD40* –1C>T was associated with the IgG response against PvDBP, whereas IgG antibody titers against PvMSP–1_19_ were influenced by the polymorphism *CD86* +1057G>A. These data may help to elucidate the immunological aspects of vivax malaria and consequently assist in the design of malaria vaccines.

## Introduction

*Plasmodium vivax* is the most prevalent species outside Africa, and *P*. *vivax* infections are responsible for the high morbidity observed in affected populations despite the lower lethality compared with infections caused by *P*. *falciparum* [1]. In endemic areas for malaria, particularly where the transmission rate is high, as age and exposure increase, subjects tend to become less susceptible to malaria episodes due to the development of an effective immune response against the parasite [2]. The role of antibodies in protection against malaria is well documented, and the passive transfer of antibodies from the serum of immune individuals to patients infected with *P*. *falciparum* effectively controls blood-stage parasites and reduces the clinical signs of the disease [3, 4]. Therefore, the development of a vaccine capable of providing protection against the blood stages of the malaria parasite will greatly decrease the clinical and economic burden of the disease.

The blood stage proteins of *Plasmodium* considered to be the main candidate targets for vaccine development include merozoite surface protein 1 (MSP–1), Duffy binding protein (DBP), and apical membrane antigen 1 (AMA–1). After two successive cleavages, only a 19 kDa C-terminal portion of MSP–1 (MSP–1_19_) remains anchored to the surface of merozoites during erythrocyte invasion, and it is believed that MSP–1_19_ is involved in the initial adhesion of merozoites to erythrocytes [5]. AMA–1 is an integral membrane protein that is essential for the reorientation of merozoites prior to erythrocyte invasion [5]. Furthermore, the binding of AMA–1 to rhoptry neck protein (RON2) is an important step in the formation of the junction complex during invasion [6]. In *P*. *vivax*, the binding of DBP to its receptor Duffy antigen receptor for chemokines (DARC) plays an important role in the binding of merozoites of this species to host reticulocytes [7]. Antibodies directed against these proteins have been shown to inhibit the binding of these proteins and prevent the invasion of erythrocytes by merozoites [8–11]. In addition, some longitudinal studies have associated AMA–1 and MSP–1_19_ antibodies with a decreased risk of malaria [12,13].

B cells require two types of signals to become activated and produce antibodies. The first signal is provided by antigen binding to the B cell receptor (BCR). Activated T cells generally provide the second signal for B cell activation through a variety of proteins. The CD40 protein is a member of the tumor necrosis factor (TNF) receptor family, which are expressed on the surface of a wide variety of cells, including B cells. The binding of CD40 to its ligand CD40L expressed on the surface of activated T cells provides the major costimulatory signal for B cells to mount a humoral response [14]. The interaction mediated by this signaling pathway is responsible for B cell proliferation and differentiation, immunoglobulin isotype switching, and antibody secretion [15,16]. Upon B cell activation, the expression of the CD86 molecule increases. In addition to the important role of this molecule in T cell activation, the binding of CD86 to its receptor, CD28, provides bidirectional signals that appear to be important for IgG production in B cells [17]. B-lymphocyte stimulator (BLyS) is a member of the TNF family present on the surface of many cells, including monocytes, macrophages, and activated T cells, or it can occur in a soluble form. Its main function is to provide signals for B cell survival and proliferation [18].

It is known that the genetic component of the host plays an important role in the development of an immune response against malaria [19]. The role of gene polymorphisms in the immune system in the production of naturally acquired antibodies has been documented in *P*. *falciparum* [20–27]. However, few studies have assessed the genetic mechanisms involved in the production of antibodies against *P*. *vivax* proteins [28–31]. Thus, this study aimed to evaluate the effects of single nucleotide polymorphisms (SNPs) in the genes *CD40*, *CD40L*, *CD86* and *BLYS* on the production of IgG antibodies against candidate vaccine proteins from *P*. *vivax* in a naturally exposed population in the Brazilian Amazon.

## Materials and Methods

### Study area and population sample

The study was conducted in the municipality of Goianésia do Pará (03°50'33 "S, 49°05'49" W), approximately 300 km from the city of Belém, capital of the state of Pará, in the Brazilian Amazon region. The climate is tropical semi-humid, with an average annual temperature of 26.3°C and average annual rainfall of approximately 2,000 mm^3^.

In this municipality, despite the seasonal rainfall pattern characterized by a dry season between June and November and a rainy season between December and May, malaria transmission is unstable and occurs throughout the year. The annual parasite incidence rates in 2011 and 2012 were 99 and 39 per 1,000 inhabitants, respectively. More than 80% of malaria cases are due to *P*. *vivax*, and the main vector in the region is *Anopheles darlingi* (Primo, unpublished data).

Samples were collected at the municipal health center between February 2011 to August 2012, and 223 individuals infected with *P*. *vivax* with classic symptoms of malaria who sought the malaria diagnostic service were recruited. In addition, 96 uninfected individuals who sought medical care offered during the study were invited to participate in the study. These participants had no close kinship and, therefore, were genetically unrelated, which was evidenced by a demographic questionnaire. Samples from 40 malaria-naive individuals residing in a non-endemic area (São José do Rio Preto, Brazil) and who never visited malaria transmission areas were used as controls.

### Blood sample collection and malaria diagnosis

After applying a questionnaire to assess demographic and epidemiological data, blood was collected in EDTA-containing test tubes, after which plasma samples were separated by centrifugation and stored at –20°C. Malaria was diagnosed using thick smears stained with Giemsa according to the malaria diagnosis guidelines of the Brazilian Ministry of Health. Subsequently, all participants (including the non-infected) had their diagnoses confirmed by nested–polymerase chain reaction (PCR) [32]. All participants or their guardians signed an informed consent form. The project was approved by the health authorities of Goianésia do Pará and by the Research Ethics Committee of the College of Medicine of São José do Rio Preto (CEP/FAMERP No. 4599/2011).

### Genotyping of the genes *CD86*, *CD40L*, *CD40*, and *BLYS*

DNA was extracted from peripheral blood samples using the Easy-DNA^™^ extraction kit (Invitrogen, California, USA). The following SNPs were identified using PCR–restriction fragment length polymorphism (RFLP): +1057G>A in *CD86* (rs1129055), –726T>C in *CD40L* (rs3092945), –1C>T in *CD40* (rs1883832), and –871C>T in *BLYS* (rs9514828). To amplify the polymorphisms in *CD40* and *CD40L*, the protocol described by Malheiros and Petz-Erler [33] was used with modifications, and amplification of the polymorphisms in *CD86* and *BLYS* followed the protocol described by Cassiano et al. [34]. Briefly, all PCR reactions were performed in a final volume of 25 μL containing 1× Buffer (20 mM Tris-HCl, 50 mM KCl, pH 8.4), 1.5 mM MgCl_2_, 0.2 mM of each dNTP, 0.4 pmol of each primer, and 0.5 U of Platinum Taq DNA Polymerase (Invitrogen, São Paulo, Brazil). Amplification was performed under the following reaction conditions: an initial step of 5 min at 94°C, 35 cycles of 30 s at 94°C, 30 s at 56°C (except for the SNP in gene *BLYS*, where the annealing temperature was 50°C) followed by 1 min at 72°C, and a final step of 10 min at 72°C. The amplification products were digested using restriction enzymes (Fermentas, Vilnius, Lithuania) according to the manufacturer's recommendations. Primer sequences and their restriction enzymes, and the restriction fragments obtained after digestion of each polymorphism are presented in S1 Table.

### Antigens

Three recombinant *P*. *vivax* proteins were used in this study. PvMSP–1_19_, corresponding to amino acids 1616–1704 of MSP–1 protein from the Belém strain, was expressed in *Escherichia coli* with a polyhistidine affinity tag (6xHis tag) [35]. A gene coding for a recombinant protein corresponding to amino acids 43–487 of the ectodomain of PvAMA–1 was synthesized by GenScript USA Inc. (Piscataway, NJ) and expressed in *Pichia pastoris* [11]. Region II of DBP of *P*. *vivax* strain Sal1 (PvDBP), which includes amino acids 243–573, was expressed in *E*. *coli* as a 6xHis fusion protein [36].

### Antibody assays

The assessment of IgG antibodies against *P*. *vivax* recombinant proteins was performed as described previously [11, 35, 36]. Briefly, the concentrations used for PvMSP–119, PvAMA–1, and PvDBP were 2 μg/mL, 2 μg/mL, and 3 μg/mL, respectively. All plasma samples were diluted at 1:100 and added in duplicate. Monoclonal antibody binding was detected using peroxidase conjugated anti-human immunoglobulin (Sigma, St Louis, USA). The results for total IgG are expressed as reactivity index (RI), which was calculated by dividing the optical density (OD) of the sample by the cut-off value, which in turn was calculated by averaging the OD values of the 40 plasma samples from the control subjects residing in the non-endemic area plus three standard deviations. Individuals with RI > 1 (also known as responders) were considered positive.

### Estimates of interethnic admixture

The population of northern Brazil is highly mixed and formed mainly by crosses between Europeans, Africans, and Native Americans. To avoid spurious interpretations resulting from population substructure, we used a panel of 48 ancestry informative markers (AIMs) to estimate the proportion of individual interethnic admixture in our sample, following a previously described protocol [37]. The Structure software version 2.3.4 was used, and three parental populations (European, African, and Native Americans) were assumed as described by Santos et al. [37]. These estimates were used as covariates in the multivariate analyses to adjust for population stratification.

### Statistical analysis

Statistical analysis was performed using the SNPassoc R package (R software version 3.1.1) [38]. Genotypic deviations from Hardy-Weinberg equilibrium were assessed using the exact test described by Wigginton et al. [39]. For univariate analysis, differences in proportions were assessed using the chi-square test, and differences between means were assessed using Student's t-test or Mann-Whitney U-test, depending on whether the data were parametric. The correlation between IgG antibody titers against PvAMA–1, PvDBP, and PvMSP-1_19_ was assessed using the Spearman correlation coefficient. The analysis of association between the SNPs and the antibody responses frequency used a logistic regression model, and the factors significantly associated with the antibody responses in the univariate analysis (gender, previous history of malaria infection, and current infection) were included as covariates. Similarly, generalized linear regression was used to assess associations between the SNPs and the magnitude of the IgG antibody responses. In all the multivariate analyses, SNPs were included following different genetic models: dominant (11 vs 12 + 22), recessive (11 + 12 vs 22), and log-additive (0, 1, 2 alleles). Values of p<0.05 were considered significant.

## Results

### Population profile

The profile of the study population is summarized in Table 1. The age of the study population varied between 14 and 68 years (median of 30 years) and the male/female sex ratio was 1.47. The period of residence in the study area varied between 0 (newly arrived migrants) to 37 years (median of 7 years). The exact period of time for which individuals had been continuously exposed to malaria could not be reliably determined because of the high rates of migration characteristic of the Brazilian Amazon population. As previously reported by Cassiano et al. [34], the population of Goianésia do Pará is highly mixed, showing a higher proportion of European genetic ancestry (44%) and significant contributions of African (31.4%) and Native American ancestry (24.6%). Most participants (78.7%) reported having had previous malaria infections, and at the time of blood collection, 69.9% (223/319) were infected with *P*. *vivax* (diagnosed by thick smear). Further analysis by nested PCR indicated that 13 of the 223 infected individuals (5.8%) were infected with both *P*. *falciparum* and *P*. *vivax*, and no individual diagnosed as negative by thick smear was positive by nested PCR.

**Table 1:** Summary of the epidemiological data and seropositivity of the study population.

		PvAMA-1			PvDBP			PvMSP-1_19_		
Characteristics	All individuals (319)^a^	Positive (164)^a^	Negative (132)^a^	p^e^	Positive (180)^a^	Negative (104)^a^	p^e^	Positive (202)^a^	Negative (89)^a^	p^e^
Gender, male (%)	59.6	68.3	47.7	0.0004	64.8	47.1	0.0004	66.8	40.4	<0.0001
Age, median years (range)	30 (14–68)	30.5 (14–68)	29 (14–66)	0.52	29 (14–68)	30.5 (14–66)	0.63	30 (14–68)	29 (15–65)	0.37
Time of residence^b^, median years (range)	7 (0.1–37)	6.0 (0.1–37)	8.5 (0.1–37)	0.06	6.5 (0.1–37)	8.0 (0.1–37)	0.16	6.5 (0.1–37)	8.5 (0.1–37)	0.15
Genetic ancestry^c^, mean ± SD (%)										
African	31.4 ± 11.0	31.3 ± 11.6	31.9 ± 10.3	0.66	31.6 ± 11.1	31.8 ± 11.3	0.92	31.6 ± 11.4	30.6 ± 9.7	0.55
European	44.0 ± 11.9	44.0 ± 11.9	43.8 ± 12.6	0.91	44.1 ± 11.8	43.6 ± 13.2	0.76	43.6 ± 12.4	45.3 ± 11.6	0.34
Native American	24.6 ± 9.4	24.6 ± 9.7	24.2 ± 9.1	0.70	24.3 ± 9.5	24.6 ± 9.5	0.77	24.8 ± 9.4	24.1 ± 9.5	0.60
Previous malaria infection^d^ (%)	78.7	95.0	55.9	<0.0001	92.0	52.2	<0.0001	90.1	49.4	<0.0001
Individuals infected with *P*. *vivax* (%)	69.9	81.7	50.0	<0.0001	78.9	44.2	<0.0001	80.2	37.1	<0.0001

^a^Number of individuals. The differences in the total number of individuals evaluated for each protein corresponding to samples that lacked plasma.

^b^Time of residence in Goianésia do Pará.

^c^Data of genetic ancestry obtained from 273 individuals.

^d^Proportion of individuals who contracted malaria in the past.

^e^P-values were calculated from a chi-squared test for qualitative variables, the Mann-Whitney test for nonparametric continuous variables and Student’s t-test for parametric continuous variables.

### Naturally acquired IgG antibodies against blood-stage proteins of *P*. *vivax*

Of the 319 participants, 296 (92.8%) had their plasma samples evaluated for IgG against PvAMA–1, 284 (89.0%) were evaluated for IgG against PvDBP, and 291 (91.2%) were evaluated for IgG against PvMSP–1_19_, which reflects the differences shown in Table 1 regarding the total number of individuals. In addition, 69.1% (202/291) of the participants had antibodies (RI > 1) against PvMSP–1_19_, 63.4% (180/284) had antibodies against PvDBP, and 55.4% (164/296) had antibodies against PvAMA–1. Among the subjects evaluated for the three proteins, 80% (223/279) had antibodies against at least one protein and 46.2% (129/279) showed responses against all three proteins. Significant positive correlations were observed between the IgG antibody titers against PvAMA–1 and PvMSP–1_19_, between PvAMA-1 and PvDBP, and between PvDBP and PvMSP–1_19_ (r = 0.72, 0.68, and 0.51, respectively, using Spearman correlation, p < 0.0001).

We assessed whether the frequency of individuals with antibodies against the proteins studied was correlated with any demographic or epidemiological variables (Table 1). The genetic ancestry proportions did not differ between subjects with or without antibodies (all p values > 0.34). Furthermore, no association was observed between the period of residence in the study area and the antibody response. However, a higher frequency of responders to all the proteins evaluated was observed among male subjects (p < 0.001). A higher proportion of individuals who had never contracted malaria was observed among those without antibodies (p < 0.0001) regardless of the protein evaluated. Furthermore, as expected, individuals who were infected at the time of blood collection showed a higher frequency of responses to all three proteins evaluated (p < 0.0001).

### Associations of B-cell co-stimulatory gene polymorphisms with the frequency of IgG responses against PvAMA-1, PvDBP, and PvMSP–1_19_

Polymorphisms in the genes studied were successfully genotyped in all samples, except for SNP rs1883832 in the *CD40* gene, which was not identified in two samples. No significant deviation from Hardy-Weinberg equilibrium was observed for any polymorphism (all p values > 0.06). The minor allele frequencies (MAF) were as follows: 0.249 for SNP rs9514828 in the *BLYS* gene (allele T), 0.216 for SNP rs1129055 in the *CD86* gene (allele A), 0.155 for SNP rs1883832 in the *CD40* gene (allele T), and 0.112 for SNP rs3092945 in the *CD40L* gene (allele C) (S2 Table). These allele frequencies were similar to those found in a previously analyzed subset of these samples [34].

**Fig 1:**
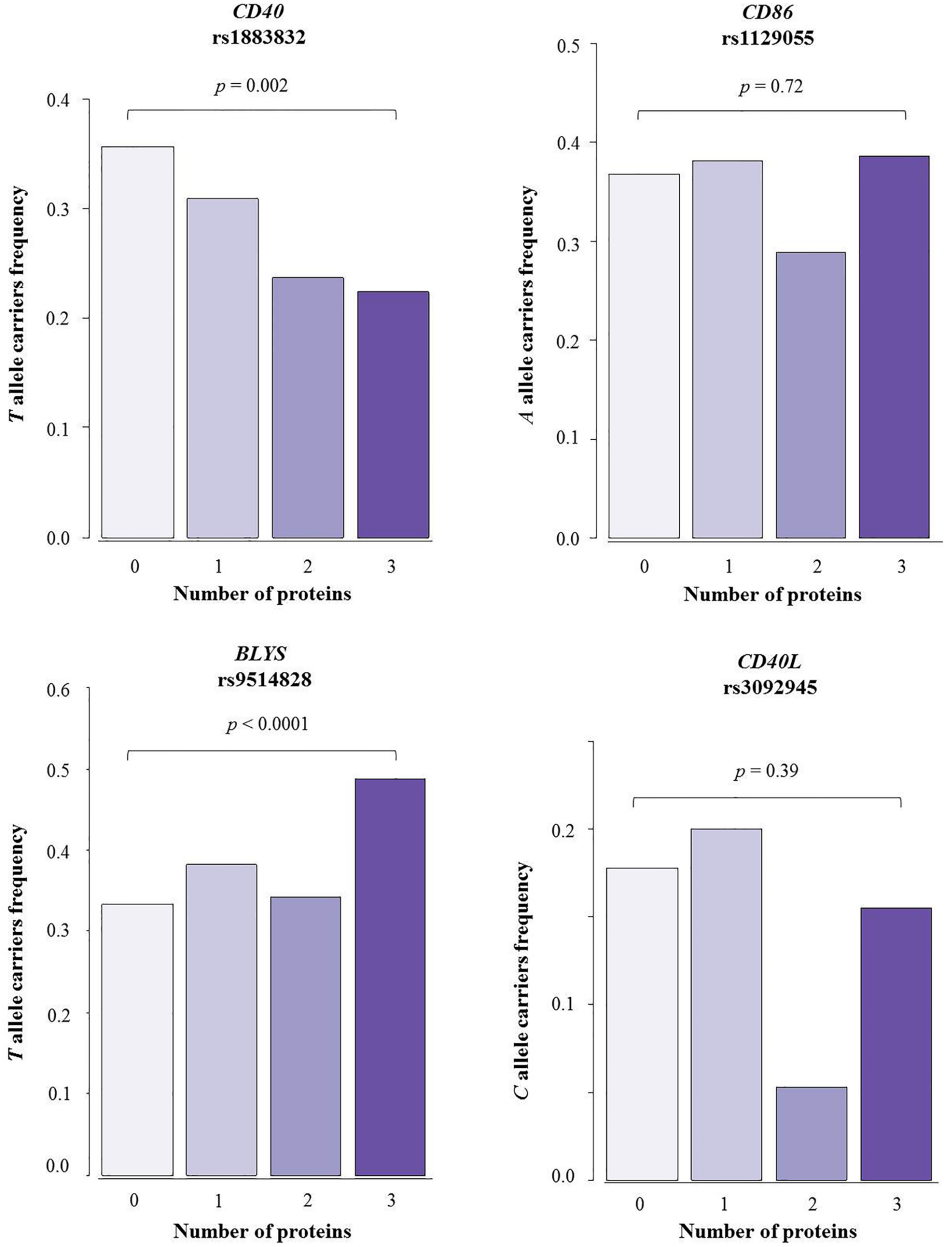
Positive antibody response and carrier frequency of mutant alleles. Frequency of carriers of mutant alleles of SNPs in genes *CD40*, *BLYS*, *CD86*, and *CD40L* according to the number of proteins for which subjects were responders. Individuals with antibodies against the three proteins (n = 279) were classified according to their reaction against zero (n = 57), one (n = 55), two (n = 38), or three (n = 129) proteins of blood-stage *P*. *vivax*.

The effects of polymorphisms in the *BLYS*, *CD86*, *CD40*, and *CD40L* genes on the IgG antibody responses against the proteins PvAMA–1, PvDBP, and PvMSP–1_19_ are shown in Table 2 and S3 Table. The additive, recessive, and dominant genetic models were tested for each SNP. With regard to PvAMA–1, the IgG antibody response was positively correlated with the presence of the T allele for SNP rs9514828 in the *BLYS* gene based on an additive model (OR = 1.59, 95% CI: 1.05–2.40; p = 0.03). The T allele for SNP rs1883832 in the *CD40* gene also followed an additive model and was negatively correlated with the IgG antibody response against PvDBP (OR = 0.57; 95% CI: 0.35–0.92, p = 0.02). Considering a dominant model, there was a greater likelihood for individuals harboring the T allele in genotypes TT and TC of SNP rs9514828 in the *BLYS* gene to have antibodies against PvMSP–1_19_ compared with individuals who harbored the CC genotype (OR = 2.01; CI: 1.12–3.61, p = 0.01). An analysis of interactions between the polymorphisms produced no additional information beyond the information obtained by the individual analysis of the polymorphisms (data not shown). In addition, we evaluated the distribution of genotypes/alleles according to the number of proteins for which individuals had antibodies, i.e., whether they responded against one, two, three, or no proteins (Fig 1). It was observed that the frequency of carriers of the T allele of SNP rs1883832 in the *CD40* gene progressively decreased as the antibody response increased (χ^2^ = 9.01; p = 0.002, Chi-squared for trends). In addition, the presence of allele *T* from SNP rs9514828 in gene *BLYS* was higher among the individuals who responded against all proteins tested (χ^2^ = 25.30; p < 0.0001, Chi-square for trends).

**Table 2:** Associations between Polymorphisms and Antibody Responses against Blood-Stage Proteins of *P*. *vivax*.

				PvAMA-1		PvDBP		PvMSP-1_19_	
Gene	SNP	Model	Genotype	OR (95%CI)^a^	p^b^	OR (95%CI)^a^	p^b^	OR (95%CI)^a^	p^b^
*BLYS*	rs9514828	Dominant	*C/C*	1.00	**0.04**	1.00	0.39	1.00	**0.01**
			*C/T–T/T*	1.68 (1.01–2.79)		1.26 (0.74–2.14)		2.01 (1.12–3.61)	
		Recessive	*C/C–C/T*	1.00	0.22	1.00	0.80	1.00	0.83
			*T/T*	1.91 (0.65–5.57)		0.87 (0.30–2.51)		0.89 (0.29–2.74)	
		Log-Additive	*0*,*1*,*2*	1.59 (1.05–2.40)	**0.03**	1.14 (0.74–1.74)	0.56	1.47 (0.91–2.37)	0.11
*CD86*	rs1129055	Dominant	*G/G*	1.00	0.85	1.00	0.45	1.00	0.22
			*G/A–A/A*	0.95 (0.57–1.58)		1.23 (0.72–2.11)		0.70 (0.39–1.24)	
		Recessive	*G/G–G/A*	1.00	0.61	1.00	0.17	1.00	0.31
			*A/A*	1.32 (0.45–3.85)		2.19 (0.69–6.96)		1.84 (0.56–6.07)	
		Log-Additive	*0*,*1*,*2*	1.01 (0.67–1.52)	0.97	1.28 (0.83–1.97)	0.26	0.88 (0.56–1.38)	0.58
*CD40*	rs1883832	Dominant	*C/C*	1.00	0.36	1.00	**0.03**	1.00	0.31
			*C/T–T/T*	0.77 (0.44–1.35)		0.53 (0.30–0.94)		0.73 (0.39–1.35)	
		Recessive	*C/C–C/T*	1.00	0.66	1.00	0.21	1.00	0.92
			*T/T*	1.35 (0.35–5.27)		0.39 (0.09–1.66)		1.09 (0.23–5.13)	
		Log-Additive	*0*,*1*,*2*	0.87 (0.55–1.37)	0.54	0.57 (0.35–0.92)	**0.02**	0.81 (0.48–1.35)	0.42
*CD40L*^c^	rs3092945	Dominant	*T/T*	1.00	0.63	1.00	0.36	1.00	0.70
			*T/C–C/C*	1.24 (0.51–3.06)		1.54 (0.61–3.88)		0.83 (0.31–2.18)	
		Recessive	*T/T–T/C*	1.00	0.70	1.00	0.60	1.00	0.92
			*C/C*	1.46 (0.21–10.39)		0.59 (0.09–4.03)		0.89 (0.08–9.86)	
		Log-Additive	*0*,*1*,*2*	1.21 (0.59–2.48)	0.60	1.24 (0.58–2.64)	0.58	0.86 (0.38–1.94)	0.72

^a^OR stands for odd ratio and CI stands for confidence intervals.

^b^p values based on fitting logistic regression models adjusted for gender and current malaria infection. P values < 0.05 are in bold.

^c^Genotypes available only for women because the *CD40L* gene is located on chromosome X.

### Associations of polymorphisms with IgG antibody titers against PvAMA–1, PvDBP, and PvMSP–1_19_

**Fig 2:**
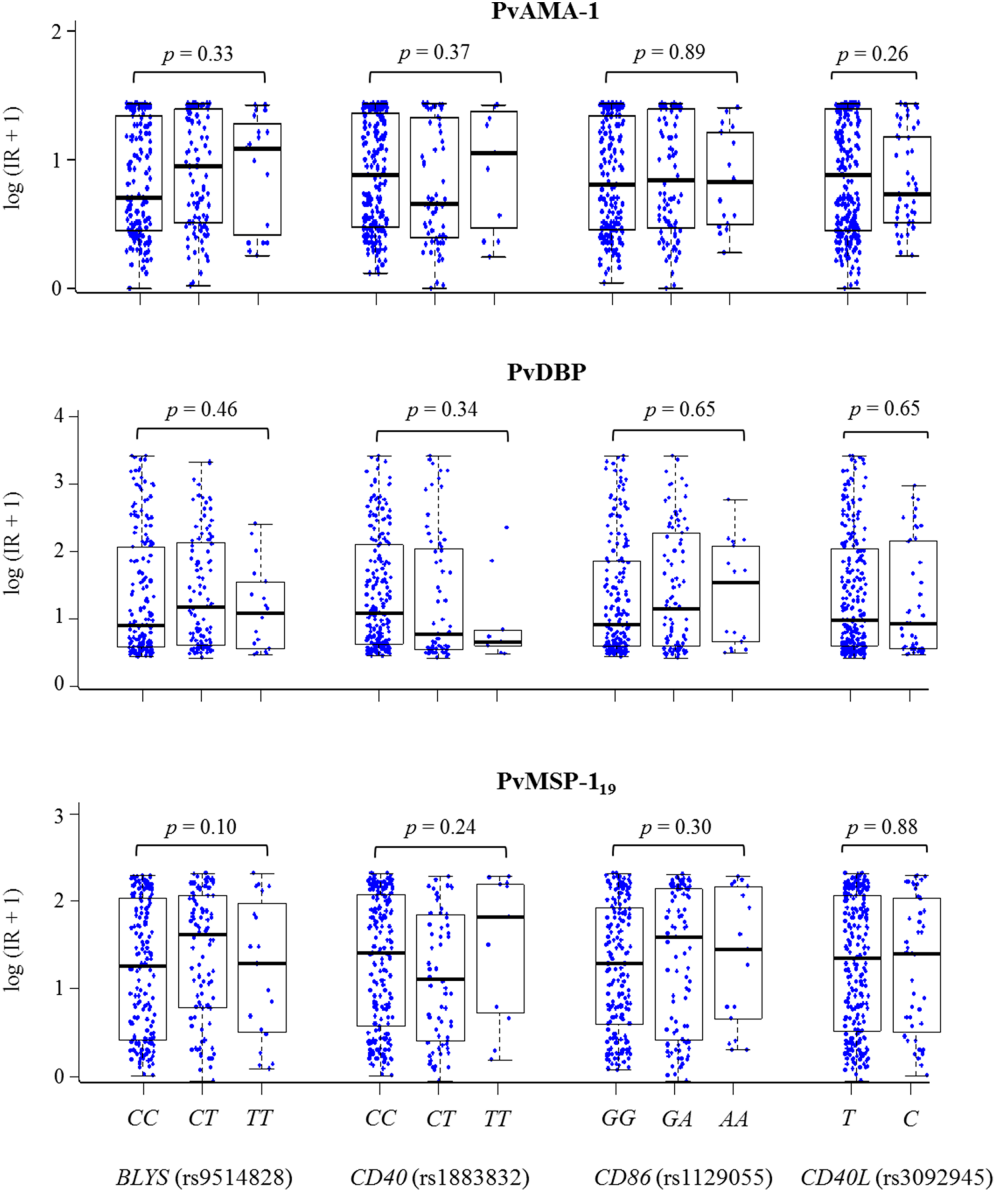
*BLYS*, *CD40*, *CD86*, and *CD40L* genotypes in relation to antibody titers against the merozoite proteins. Antibody titers were expressed as log-transformed reactivity indices (RI). For the SNP in the gene *CD40L*, men and women harboring the C allele were grouped and compared with individuals who did not possess this allele given that *CD40L* is located on chromosome X. Multivariate logistic regression analysis found no significant differences in the antibody titers between the different genotypes.

We evaluated the effects of the polymorphism on IgG antibody titers against the three *P*. *vivax* proteins. The variables that affected antibody titers, corresponding to the same variables associated with response frequency (gender, past history of malaria infection, and current infection) (S4 Table), were included in the multivariate analysis together with the polymorphisms. There was no significant effect of any genotype/allele investigated on the antibody titers (Fig 2). However, when individuals with or without a *P*. *vivax* infection at the time of blood collection were analyzed separately, a significant increase in IgG antibody titers against PvMSP–1_19_ was observed among infected individuals harboring genotype AA of the *CD86* polymorphism compared to those harboring the GG and GA genotypes (median [Q1–Q3]: 7.87 [4.74–8.40] vs. 5.05 [1.56–7.60]; p = 0.03).

## Discussion

The present study aimed to evaluate the effects of polymorphisms in co-stimulatory genes of B cells on antibody responses against recombinant proteins of *P*. *vivax* in a population sample from the Brazilian Amazon. Although the number of studies aimed at identifying genetic mechanisms involved in regulating the production of antimalarial antibodies has increased in recent years [20–31], the development of an effective vaccine will most likely require a thorough understanding of the host–parasite relationship. Therefore, studies such as the one reported herein will help to elucidate the responses of individuals to certain vaccine protein candidates.

Importantly, in this study, potential biases due to population substructure were considered. The frequencies of the alleles *CD86* +1057A and *CD40L* –726T are higher among individuals with higher proportions of European ancestry [34], and consequently, if individuals with antibodies exhibited more European ancestry than non-responders, a false association between these alleles and antibody production could be found. However, the fact that no differences were observed in the proportion of genetic ancestry between the responders and non-responders for the three proteins studied and our inclusion of individual ancestry values as covariates in the multivariate analyses precluded the possibility of false associations in our results.

Differences were observed in the frequency of responders against the three proteins evaluated, and the proportion of responders against PvMSP–1_19_ was the highest (69.1%), followed by responders against PvDBP (63.4%), and those against PvAMA–1 (55.4%). The higher proportion of responders against PvMSP–1_19_ was an expected result considering that previous studies have shown that this protein is highly immunogenic [40, 41], most likely owing to the low degree of polymorphism found in the MSP–1 region [42], and also because MSP–1_19_ is carried into the infected cell, persists until the end of the intracellular cycle, accumulates in the digestive vacuole, and is discarded together with digestion products, possibly increasing its exposure to the immune system [43]. Both the frequency of individuals with antibodies and the magnitude of the IgG response were significantly higher in individuals who had had previous episodes of malaria and/or who were infected at the time of blood collection, suggesting the occurrence of a boosting effect in the antibody responses directed against these proteins compared with individuals who never contracted malaria. In addition, it was noted that the IgG response against the three proteins was higher in men than women. Although the reasons for this result remain unclear [29], men are most likely more exposed to malaria transmission from working for longer periods in the field compared to women.

The main result found in our study was that polymorphisms in the genes *CD40* and *BLYS* seem to influence the IgG antibody responses against PvAMA–1, PvDBP, and PvMSP–1_19_ of *P*. *vivax* in the population studied. Considering that the binding of CD40 to its ligand CD40L is critical for the production of antibodies in B cells [14], it is possible that this molecule is involved in the immune response against malaria. Indeed, this co-stimulatory pathway is important for the production of IgG antibodies against *P*. *falciparum* proteins because PBMCs from individuals living in holo- or meso-endemic malaria areas were found to produce more antibodies *in vitro* when CD40L costimulation was provided [44]. In our study, the presence of the T allele of SNP rs1883832 in the *CD40* gene was negatively correlated with the production of IgG antibodies against PvDBP. This polymorphism is located at position –1 of the start codon and affects the Kozak sequence, which is crucial to the initiation of the protein translation process [45], and it is believed that the presence of the T allele can decrease gene expression by 15%–30% [46]. Whether the correlation between the SNP in the *CD40* gene and the production of IgG antibodies against PvDBP is associated with clinical immunity requires further investigation. However, it is noteworthy that the frequency of the T allele is significantly lower in African populations than in European populations [47], and this frequency may suggest a selective advantage of the C allele due to pressure exerted by malaria. However, in a case-control study conducted in the city of Macapá in the Brazilian Amazon, no association was found between polymorphism in the *CD40* gene and susceptibility to vivax malaria [48]. This same study evaluated the influence of *BLYS*, *CD40*, and *CD40L* polymorphisms on the antibody response against recombinant proteins of *P*. *vivax* in a subgroup whose serological results were available, and the results indicated no correlation with antibody response. However, it is of note that this subgroup consisted of approximately 50 individuals. Moreover, it remains possible that the conditions of malaria transmission and the genetic background of the populations differ between the municipalities of Macapá and Goianésia do Pará, which could explain the differences observed [48].

BLyS is a critical cytokine for B cell survival and differentiation; its serum levels were found to be higher among children with acute malaria and were positively correlated with the levels of IL-10 and IFN-γ [49]. A study conducted by Liu et al. [50] demonstrated that the production of memory B cells in response to vaccination with MSP–1_19_ from *P*. *yoelli* in mice was dependent on the production of BLyS by dendritic cells (DCs). In our study, the presence of the T allele of SNP rs9514828 in the *BLYS* gene was positively correlated with the production of IgG antibodies against PvAMA–1 and PvMSP–1_19_. This SNP is located in the promoter region of the gene at position -871 relative to the start codon, corresponding to a binding site for the transcription factor MZF1, and it can therefore modulate gene expression [51]. However, the role of this SNP in gene expression has not been elucidated—some studies have associated the T allele with higher levels of *BLYS* mRNA [51,52], whereas others have not established this association [53].

It is of interest that the observed associations involving these two SNPs could not be extrapolated to the antibody response against all proteins tested, i.e., although SNP *BLYS* rs9514828 was associated with the response of IgG antibodies against PvAMA-1 and PvMSP-1_19_, no association involving this SNP and PvDBP was found. Similarly, SNP *CD40* rs1883832 was associated with PvDBP but not with PvAMA-1 and PvMSP-1_19_. Although the reasons for these results are unknown, they are likely to reflect intrinsic differences among these proteins, including the degree of polymorphism, exposure to the immune system, and antigen presentation via HLA, among others. However, as shown in Fig 1, there was a higher frequency of allele *T* carriers of SNP *BLYS* rs9514828 among those individuals who had antibodies against all three proteins evaluated, whereas the frequency of allele *T* carriers of SNP *CD40* rs1883832 was lower among the responders of all proteins.

In addition, we found a correlation between SNP rs1129055 in the *CD86* gene and the magnitude of the IgG response against PvMSP-119, but only among individuals infected with *P*. *vivax*. In murine models of malaria, the CD86 molecule seems to be involved in the differentiation of the Th2-type response [54]. Furthermore, for gene *CD28*, which is the receptor of ligand CD86, was observed a lower production of IgG antibodies against AMA-1 and MSP-1 when *CD28* knockout mice were infected with *P*. *chabaudi* [55]. In our study, we found that individuals infected with *P*. *vivax* harboring the AA genotype had the highest antibody titers against PvMSP-1_19_. The SNP rs1129055 is located in exon 8 of the gene and causes a non-silent substitution of alanine for threonine at amino acid position 304 of the protein, introducing a potential phosphorylation site in the cytoplasmic region of the molecule [56]. Although the functional implications of this polymorphism are not yet elucidated, we speculate that individuals with the *AA* genotype infected with *P*. *vivax* may have a response directed more towards the Th2-type, with increased production of antibodies, particularly against PvMSP-1_19_.

In conclusion, we found evidence for the role of co-stimulatory B cell molecules in the genetic control of the immune response against *P*. *vivax*. The identification of individual genetic traits that influence the development of an immune response may be important in the development of vaccines against malaria. However, further investigations involving these genes are necessary to confirm whether their effect on antibody production is associated with the control of *P*. *vivax* infection.

## Supporting Information

S1 Table Reaction conditions for the amplification and enzymatic digestion of polymorphisms in the genes *CD40*, *CD40L*, *BLYS*, and *CD86*. (DOCX)

S2 Table Minor Allele Frequencies of Polymorphisms in Genes *CD40*, *CD40L*, *BLYS*, and *CD86*. (DOCX)

S3 Table Polymorphism Distribution between the Groups with and without Antibodies against Blood-Stage Proteins of *P*. *vivax*. (DOCX)

S4 Table Antibody levels (RI) for PvAMA-1, PvDBP, and PvMSP-1_19_ according to gender, current infection status, and previous episode of malaria. (DOCX)
