# Supplementary material for: Polymorphisms in B Cell Co-Stimulatory Genes Are Associated with IgG Antibody Responses against Blood–Stage Proteins of Plasmodium vivax
Source: PLoS One. 2016 Feb 22;11(2):e0149581. doi: 10.1371/journal.pone.0149581 (PMC4763038; doi:10.1371/journal.pone.0149581)
Supplement: S1 Table — (DOCX) [file pone.0149581.s001.docx]

**S1 Table. Reaction conditions for the amplification and enzyme digestion of polymorphisms in the genes *CD40*, *CD40L*, *BLYS*, and *CD86*.**

| **SNP** | **Gene** | **Primer5’-3’ (forward)** | **Primer5’-3’ (reverse)** | **Restriction enzyme** | **Fragment length(bp)** |
| --- | --- | --- | --- | --- | --- |
| rs1129055 | *CD86* | CTGTTCCAATGGCAACCTCT | GGTTGCCCAGGAACTTACAA | *CviK*I-1 | G: 79 + 75 + 58 + 54, A: 154 + 58 + 54 |
| rs3092945 | *CD40L* | ATCTTCACAGCAACCTAC | CACTAAACTCAATGAAAGCC | *Lwe*I | T: 251 + 195, C: 446 |
| rs1883832 | *CD40* | GAAACTCCTGCGCGGTGAAT | GAAACTCCTGCGCGGTGAAT | *Sty*I | C: 133 + 96 + 74, T: 207 + 96 |
| rs9514828 | *BLYS* | TGGCTCTTGTGTGATCAAGG | GCCTGGTCTCAGCTTTTCTG | *Mbi*I | C: 162 + 48, T: 210 |
