# Supplementary material for: Polymorphisms in B Cell Co-Stimulatory Genes Are Associated with IgG Antibody Responses against Blood–Stage Proteins of Plasmodium vivax
Source: PLoS One. 2016 Feb 22;11(2):e0149581. doi: 10.1371/journal.pone.0149581 (PMC4763038; doi:10.1371/journal.pone.0149581)
Supplement: S2 Table — (DOCX) [file pone.0149581.s002.docx]

| **SNP** | **Gene** | **Allele** | **MAF** |
| --- | --- | --- | --- |
| rs1129055 | *CD86* | *A* | 0.216 |
| rs3092945 | *CD40L* | *C* | 0.112 |
| rs1883832 | *CD40* | *T* | 0.155 |
| rs9514828 | *BLYS* | *T* | 0.249 |

**S2 Table. Minor Allele Frequencies of Polymorphisms in Genes *CD40*, *CD40L*, *BLYS*, and *CD86.***
