# Supplementary material for: Polymorphisms in B Cell Co-Stimulatory Genes Are Associated with IgG Antibody Responses against Blood–Stage Proteins of Plasmodium vivax
Source: PLoS One. 2016 Feb 22;11(2):e0149581. doi: 10.1371/journal.pone.0149581 (PMC4763038; doi:10.1371/journal.pone.0149581)
Supplement: S3 Table — (DOCX) [file pone.0149581.s003.docx]

**S3 Table.** **Polymorphism distribution between the groups with and without antibodies against blood-stage proteins of *P. vivax*.**

|  |  | **PvAMA-1** | | | |  | **PvDBP** | | | |  | **PvMSP-1_19_** | | | |
| --- | --- | --- | --- | --- | --- | --- | --- | --- | --- | --- | --- | --- | --- | --- | --- |
| **Gene** | **SNP** | Negative  n (%) | Positive  n (%) | OR (95%CI)^a^ | p^b^ |  | Negative  n (%) | Positive  n (%) | OR (95%CI)^a^ | p^b^ |  | Negative  n (%) | Positive  n (%) | OR (95%CI)^a^ | p^b^ |
|  |  |  |  |  |  |  |  |  |  |  |  |  |  |  |  |
| *BLYS* | rs9514828 |  |  |  |  |  |  |  |  |  |  |  |  |  |  |
|  | Genotype |  |  |  | 0.11 |  |  |  |  | 0.54 |  |  |  |  | 0.06 |
|  | *C/C* | 87 (65.9) | 89 (54.3) | 1.00 |  |  | 65 (62.5) | 102 (56.7) | 1.00 |  |  | 61 (68.5) | 111 (55.0) | 1.00 |  |
|  | *C/T* | 39 (29.5) | 62 (37.8) | 1.55 (0.94-2.56) |  |  | 32 (30.8) | 67 (37.2) | 1.33 (0.79-2.25) |  |  | 22 (24.7) | 78 (38.6) | 1.95 (1.11-3.43) |  |
|  | *T/T* | 6 (4.5) | 13 (7.9) | 2.12 (0.77-5.82) |  |  | 7 (6.7) | 11 (6.1) | 1.00 (0.37-2.71) |  |  | 6 (6.7) | 13 (6.4) | 1.19 (0.43-3.29) |  |
|  | Allele |  |  |  | **0.04** |  |  |  |  | 1 |  |  |  |  | 0.10 |
|  | *C* | 213 (80.7) | 240 (73.2) | 1.00 |  |  | 162 (77.9) | 307 (77.5) | 1.00 |  |  | 144 (80.9) | 300 (74.2) | 1.00 |  |
|  | *T* | 51 (19.3) | 88 (26.8) | 1.53 (1.04-2.26) |  |  | 46 (22.1) | 89 (22.5) | 1.02 (0.68-1.53) |  |  | 34 (19.1) | 104 (25.8) | 1.47 (0.95-2.27) |  |
| *CD86* | rs1129055 |  |  |  |  |  |  |  |  |  |  |  |  |  |  |
|  | Genotype |  |  |  | 0.96 |  |  |  |  | 0.58 |  |  |  |  | 0.69 |
|  | *G/G* | 83 (62.9) | 102 (62.2) | 1.00 |  |  | 69 (66.3) | 109 (60.6) | 1.00 |  |  | 53 (59.6) | 130 (64.4) | 1.00 |  |
|  | *G/A* | 41 (31.1) | 53 (32.3) | 1.05 (0.64-1.73) |  |  | 30 (28.8) | 59 (32.8) | 1.24 (0.73-2.12) |  |  | 31 (34.8) | 60 (29.7) | 0.79 (0.46-1.35) |  |
|  | *A/A* | 8 (6.1) | 9 (5.5) | 0.92 (0.34-2.48) |  |  | 5 (4.8) | 12 (6.7) | 1.52 (0.51-4.50) |  |  | 5 (5.6) | 12 (5.9) | 0.98 (0.33-2.91) |  |
|  | Allele |  |  |  | 0.93 |  |  |  |  | 0.34 |  |  |  |  | 0.62 |
|  | *G* | 207 (78.4) | 257 (78.4) | 1.00 |  |  | 168 (80.8) | 277 (76.9) | 1.00 |  |  | 137 (77.0) | 320 (79.2) | 1.00 |  |
|  | *A* | 57 (21.6) | 71 (21.6) | 1.00 (0.68-1.49) |  |  | 40 (19.2) | 83 (23.1) | 1.26 (0.82-1.92) |  |  | 41 (23.0) | 84 (20.8) | 0.88 (0.57-1.34) |  |
| *CD40* | rs1883832 |  |  |  |  |  |  |  |  |  |  |  |  |  |  |
|  | Genotype |  |  |  | 0.32 |  |  |  |  | 0.06 |  |  |  |  | 0.33 |
|  | *C/C* | 92 (70.2) | 125 (76.2) | 1.00 |  |  | 67 (65.0) | 140 (77.8) | 1.00 |  |  | 60 (68.2) | 153 (75.7) | 1.00 |  |
|  | *C/T* | 35 (26.7) | 32 (19.5) | 0.67 (0.39-1.17) |  |  | 31 (30.1) | 36 (20.0) | 0.56 (0.32-0.97) |  |  | 25 (28.4) | 48 (20.3) | 0.64 (0.36-1.15) |  |
|  | *T/T* | 4 (3.1) | 7 (4.3) | 1.29 (0.37-4.53) |  |  | 5 (4.9) | 4 (2.2) | 0.38 (0.10-1.47) |  |  | 3 (3.4) | 8 (4.0) | 1.05 (0.27-4.07) |  |
|  | Allele |  |  |  | 0.49 |  |  |  |  | **0.02** |  |  |  |  | 0.34 |
|  | *C* | 219 (83.6) | 282 (86.0) | 1.00 |  |  | 165 (80.1) | 316 (87.8) | 1.00 |  |  | 145 (82.4) | 347 (85.9) | 1.00 |  |
|  | *T* | 43 (16.4) | 46 (14.0) | 0.83 (0.53-1.30) |  |  | 41 (19.9) | 44 (12.2) | 0.56 (0.35-0.89) |  |  | 31 (17.6) | 57 (14.1) | 0.77 (0.48-1.24) |  |
| *CD40L*^c^ | rs3092945 |  |  |  |  |  |  |  |  |  |  |  |  |  |  |
|  | Genotype |  |  |  | 0.65 |  |  |  |  | 0.50 |  |  |  |  | 0.72 |
|  | *T/T* | 61 (80.3) | 42 (75.0) | 1.00 |  |  | 48 (82.8) | 52 (74.3) | 1.00 |  |  | 44 (78.6) | 58 (78.4) | 1.00 |  |
|  | *T/C* | 13 (17.1) | 11 (19.6) | 1.23 (0.50-3.00) |  |  | 8 (13.8) | 15 (21.4) | 1.73 (0.67-4.45) |  |  | 11 (19.6) | 13 (17.6) | 0.90 (0.37-2.19) |  |
|  | *C/C* | 2 (2.6) | 3 (5.4) | 2.18 (0.35-13.61) |  |  | 2 (3.4) | 3 (4.3) | 1.38 (0.22-8.65) |  |  | 1 (1.8) | 3 (4.1) | 2.28 (0.23-22.63) |  |
|  | Allele |  |  |  | 0.94 |  |  |  |  | 0.92 |  |  |  |  | 0.99 |
|  | *T* | 183 (88.4) | 194 (88.2) | 1.00 |  |  | 142 (88.2) | 221 (88.4) | 1.00 |  |  | 128 (88.9) | 244 (88.4) | 1.00 |  |
|  | *C* | 24 (11.6) | 26 (11.8) | 1.02 (0.57-1.84) |  |  | 19 (11.8) | 29 (11.6) | 0.98 (0.53-1.81) |  |  | 16 (11.1) | 32 (11.6) | 1.05 (0.55-1.98) |  |

^a^OR stands for odd ratio and CI stands for confidence intervals.

^b^p values based on Chi-square test. P values < 0.05 are in bold.

^c^Genotypes available only for women because the *CD40L* gene is located on chromosome X.
