# Supplementary material for: Polymorphisms in B Cell Co-Stimulatory Genes Are Associated with IgG Antibody Responses against Blood–Stage Proteins of Plasmodium vivax
Source: PLoS One. 2016 Feb 22;11(2):e0149581. doi: 10.1371/journal.pone.0149581 (PMC4763038; doi:10.1371/journal.pone.0149581)
Supplement: S4 Table — (DOCX) [file pone.0149581.s004.docx]

**S4 Table.** **Antibody levels (RI) for PvAMA-1, PvDBP, and PvMSP-1_19_ according to gender, current infection status, and previous episode of malaria.**

|  | PvAMA-1 | | |  | PvDBP | | |  | PvMSP-1_19_ | | |
| --- | --- | --- | --- | --- | --- | --- | --- | --- | --- | --- | --- |
| Variable | n | RI^a^ | p^b^ |  | n | RI | p |  | n | RI | p |
| Gender |  |  | 0.01 |  |  |  | 0,01 |  |  |  | <0.0001 |
| Male | 175 | 1.71 (0.61-3.02) |  |  | 166 | 2.14 (0.88-7.50) |  |  | 172 | 3.89 (1.22-7.46) |  |
| Female | 121 | 0.91 (0.52-2.54) |  |  | 118 | 1.10 (0.72-5.13) |  |  | 119 | 1.29 (0.38-4.89) |  |
| Previous malaria infection |  |  | <0.0001 |  |  |  | <0.0001 |  |  |  | <0.0001 |
| No | 60 | 0.52 (0.40-0.75) |  |  | 57 | 0.75 (0.66-1.07) |  |  | 60 | 0.51 (0.30-1.28) |  |
| Yes | 218 | 1.96 (0.88-3.05) |  |  | 209 | 2.66 (1.04-8.29) |  |  | 213 | 4.43 (1.40-7.53) |  |
| Individuals infected with *P. vivax* |  |  | <0.0001 |  |  |  | <0.0001 |  |  |  | <0.0001 |
| No | 96 | 0.69 (0.50-1.27) |  |  | 96 | 0.85 (0.70-1.78) |  |  | 96 | 0.68 (0.35-2.09) |  |
| Yes | 200 | 2.00 (0.66-3.06) |  |  | 188 | 2.78 (1.00-8.37) |  |  | 195 | 5.09 (1.57-1.66) |  |

^a^IR expresso como mediana (Q1-Q3)

^b^Os valores de p foram calculados com o teste não paramétrico de Mann-Whitney.
